# Epidemiological trends of acute watery diarrhea (AWD) across all age groups at Cumilla General Hospital: Retrospective cross-sectional insights from AWD surveillance data of 2022 to 2024

**DOI:** 10.1016/j.ijregi.2026.100916

**Published:** 2026-05-22

**Authors:** Habib Md. Ahashan, Sarker Mohammad Ferdous Rahman, Ahmed Tanvir, Hasan Md. Rafiul, Islam Md. Taufiqul, Khan Zahid Hasan, Amin Mohammad Ashraful, Ahmed Quazi Zaki, Hossain Rakib, Rahman Urmila, Shirin Tahmina, Qadri Firdausi, Khan Ashraful Islam

**Affiliations:** 1 Mycobacterial Disease Control (MBDC), Directorate General of Health Services (DGHS), Ministry of Health and Family Welfare (MoHFW), Dhaka, Bangladesh; 2 Institute of Epidemiology, Disease Control & Research (IEDCR), Mohakhali, Dhaka, Bangladesh; 3 Communicable Disease Control (CDC), Directorate General of Health Services (DGHS), Dhaka, Bangladesh; 4 Upazila Health Complex, Patharghata, Barguna, Barishal, Bangladesh; 5 International Centre for Diarrhoeal Disease Research, Bangladesh (icddr,b), Dhaka, Bangladesh; 6 Economics Discipline, Khulna University, Khulna, Bangladesh; 7 Dr. Sirajul Islam Medical College & Hospital Ltd., Dhaka, Bangladesh

**Keywords:** Acute watery diarrhea, Bangladesh, Cholera, Epidemiology, Public health, Surveillance

## Abstract

**Objectives:**

Acute watery diarrhea (AWD) remains prevalent (16/1000 population) in Bangladesh. Nationwide AWD surveillance has been active since 2014 across 16 sentinel sites. With an estimated >100,000 cases and approximately 3,500 deaths annually, cholera remains a critical public health concern. This study analyzed AWD surveillance data from June 2022 to September 2024.

**Methods:**

Suspected cholera cases (rapid diagnostic test [RDT]-positive and culture/polymerase chain reaction [PCR]-positive for cholera) were identified using the United States Centers for Disease Control and Prevention (CDC) clinical definitions, RDTs, and microbiological culture. Socio-demographic, clinical, and etiological characteristics were examined descriptively. Cases identified using the Cholkit RDT.

**Results:**

Among 977 cases, 49 (5%) were RDT-positive for cholera. Culture was not conducted in 41/49 (84%) cases. Children aged <5 years were predominantly affected (56.1%). Two culture-positive cases were RDT-negative. Suspected case surges occurred in November and January. All cases reported drinking untreated water and not using soap after defecation; 67.45% consumed roadside food. Alarmingly, 90% (881/977) of suspected cases had taken antibiotics before hospital admission. The Cumilla Adarsha Sadar sub-district bore the highest burden (66.8% of suspected cases). RDT-positive cases showed variation across demographic and clinical characteristics.

**Conclusion:**

The study indicates a substantial burden of AWD, with a small proportion of cases testing RDT-positive for cholera. Enhancing diagnostic capacity particularly microbiological culture use is essential for accurate detection. Public health efforts must prioritize promotion of safe water and handwashing practices.

## Highlights

• Acute watery diarrhea (AWD) burden is high; only ∼5% cases were positive for cholera via rapid diagnostic testing.
• Children aged <5 years accounted for most AWD cases in surveillance.
• High antibiotic use (90%) were observed before hospital admission.
• Seasonal peaks seen in monsoon and post-monsoon periods.
• Limited culture testing restricts accurate cholera confirmation.

## Introduction

Acute watery diarrhea (AWD) is primarily caused by infectious agents such as *Vibrio cholerae*, rotavirus, and enterotoxigenic Escherichia coli, which lead to severe clinical manifestations including dehydration and electrolyte imbalance [1], [2]. AWD remains prevalent (16/1000 population) in Bangladesh [3]. The biological mechanism involves the production of enterotoxins that disrupt intestinal absorption, resulting in rapid fluid loss 4. AWD is a major contributor to morbidity and mortality globally, particularly in low- and middle-income countries. Globally, approximately 1.7 billion childhood diarrheal episodes occur each year and it is one of the top five causes of mortality among children aged <5 years [5], [6],. In Bangladesh, AWD remains a persistent public health problem, with seasonal peaks and increases following climate-related events such as floods. Cholera and other diarrheal pathogens pose recurring epidemics in certain densely populated urban slum areas despite the overall improvement of healthcare and sanitary condition. These settings are usually characterized by limited water, sanitation, and hygiene (WASH) facilities that expose vulnerable populations to AWD. Further, since the threat of antimicrobial resistance (AMR) and dynamic epidemiology continues to change, tracking surveillance and evidence-based response is vital. In such settings, contamination may also occur at the system level, including potential leakage between sewage and drinking water pipelines, which can compromise water safety irrespective of household hygiene practices.

An estimated 66 million people in Bangladesh are prone to cholera, where about 109,052 cases are reported annually that cause nearly 3500 deaths [7]. Pressure on the health care system is tremendous, as hospitals such as Cumilla General Hospital are often filled to capacity during peak seasons and both resources and quality of care suffer from the overload. The disease is known to exhibit high seasonality in Bangladesh during the monsoon and post-monsoon periods because flooding leads to the contamination of the water supply [8], [9]. Studies in Bangladesh and South Asia have consistently connected AWD outbreaks to contamination of drinking water sources, inadequate sanitation, open defecation, and absence of hand hygiene especially among low-income urban and rural resources [3], [10]. AWD and cholera outbreaks in Bangladesh have usually occurred after natural disasters or because of behavioral risk reasons. The largest outbreaks of diarrheal diseases occurred during the floods of 1988, 1998, and 2004 11. More recently, in 2021, AWD was reported from 11 areas, such as Bhola, Barishal, Patuakhali, Pirojpur, and Bandarban [12]. Despite the existence of surveillance systems, AWD outbreaks continue to exert substantial health and economic burdens in Bangladesh. Key challenges include delays in early detection, limited diagnostic capabilities, and the lack of comprehensive community-level data all of which hinder timely public health responses. Recent cholera outbreaks following extreme climatic events highlight the vulnerability of health systems and water sanitation infrastructure in affected regions [13].

The prevailing model in Bangladesh is passive hospital-based surveillance, but it underestimates the true extent of AWD on account of low health care access and utilization among the most marginalized [14]. The 2022-2024 AWD surveillance data highlighted the immediate requirement of reinforcing surveillance mechanisms and adaptation action to address future outbreaks appropriately. Reflections from AWD outbreaks in conflict and humanitarian settings draw attention to the necessity for public health response that is adaptive and sensitive to displacement, fragile healthcare systems, and provision of basic services [15]. This study aims to identify outbreaks, assess intervention effectiveness, and guide public health strategies. It explores demographic, clinical, and environmental factors of AWD in Cumilla, addressing not only clinical concerns but also economic, social, and policy aspects. By filling an evidence gap, it offers critical insights to improve surveillance, response, and policy, supporting data-driven actions to reduce diarrheal burden and build community resilience amid rising water insecurity from climate change.

## Materials and methods

This study was carried out at Cumilla General Hospital, which is one of the sentinel sites on Bangladesh’s national AWD surveillance network. Data was captured from patients during June 2022-September 2024. The study population consisted of subjects of all ages who met the clinical case definition and required hospitalization. Selection criteria excluded patients with AWD lasting more than 14 days, those with bloody diarrhea, or those diagnosed with inflammatory bowel disease or medication-induced diarrhea.

Cholera testing was conducted using rapid diagnostic tests (RDTs) on stool specimens collected from suspected AWD cases. Stool samples were collected in sterile containers and tested immediately or within a short time frame to maintain sample integrity. Quality control procedures included adherence to standard operating protocols and periodic supervision by trained laboratory personnel. All RDTs were performed by trained healthcare staff at the sentinel surveillance site. Because of resource constraints, microbiological culture and polymerase chain reaction (PCR) confirmation were not routinely conducted.

Data were drawn from both aggregated monthly records and individual case-report forms (CRFs). Convenience sampling was used, and data collection was carried out through face-to-face interviews using structured CRFs after obtaining written informed consent. The final dataset comprised 977 cases, exceeding the initially calculated sample size of 330, which accounted for an estimated cholera prevalence of 20% and a 25% allowance for missing data. Cases identified using the Cholkit RDT. Because of limited variability in certain environmental and behavioral variables (e.g. water treatment and handwashing practices), these factors were not suitable for meaningful comparative risk analysis and were interpreted descriptively.

### *Operational definitions/Case definitions of the study*

1. *AWD:*
  AWD is defined as a patient presenting with the sudden onset of ≥3 loose or watery stools within a 24-hour period. The illness typically occurs within the past 14 days. Additional symptoms may include abdominal cramps, nausea, vomiting, and fever, although these are not always present. Epidemiological links may involve recent travel to areas with known AWD outbreaks or exposure to contaminated water or food sources or contact with individuals showing similar symptoms. Signs of dehydration such as dry mouth, decreased urine output, dizziness, or lethargy may also be present.
2. *Suspected case of cholera:*
  A suspected cholera case involves a patient with AWD (≥3 episodes in 24 hours) and a history of travel to a cholera-affected area or known contact with a RDT-positive cholera case or culture-positive cholera case.
3. *Rapid diagnostic test-positive cholera case or culture/polymerase chain reaction-positive cholera:*
  **RDT-positive cholera case:** An RDT-positive cholera case is defined as a patient presenting with AWD who tests positive for *Vibrio cholerae* using a RDT on a stool sample.
  **Culture/PCR-positive cholera case:** A culture/PCR-positive cholera case is defined as a patient in whom *Vibrio cholerae* is identified in a clinical specimen (e.g. stool, vomit, or rectal swab) through laboratory confirmation using microbiological culture or PCR.

**Diagnostic approach in Bangladesh:**

In Bangladesh, confirmation of cholera primarily relies on laboratory identification of *Vibrio cholerae* from stool specimens. Commonly used methods include:

**Culture:** Stool samples are enriched in alkaline peptone water and plated on thiosulfate-citrate-bile salts-sucrose (TCBS) agar, where *Vibrio cholerae* appear as yellow colonies because of sucrose fermentation.
**Molecular methods:** PCR assays are also used for rapid detection, especially during outbreaks, offering timely confirmation of cholera cases.
**RDT:** A RDT for cholera is a quick, field-based diagnostic tool used to detect the presence of *Vibrio cholerae,* the bacterium responsible for cholera in stool samples.

### *Laboratory testing*

The surveillance employed a passive, indicator-based reporting system, with diagnostic confirmation based on RDTs. Microbiological culture or PCR was not routinely conducted because of resource constraints.

### *Statistical analysis*

Key variables included demographic information, clinical symptoms, risk exposures, and geographic distribution. Data was cleaned and recorded using Microsoft Excel and analyzed with STATA 17. Descriptive statistics summarized demographic and clinical features, while associations between variables and RDT positivity were assessed using chi-square tests. Effect estimates are presented with corresponding 95% confidence intervals and exact *P*-values.

## Results

### Socio-demographic characteristics

Among 977 suspected AWD cases tested, 49 (5.01%) were RDT-positive. Children aged <5 years made up most suspected cases (56.1%) but a smaller share of positives (44.9%), while adults aged 18-45 years were more represented among positives (38.8% vs. 27.1%), although the estimate suggested a higher proportion among positives. Gender distribution was similar across groups. Among adults, housewives were most common (∼59% in both groups); service holders were more frequent among positives (16.7% vs. 8.8%). Notably, RDT-positive adults had lower rates of no formal education (16.7% vs. 38.8%) and higher rates of secondary or higher education, with the estimate suggesting a potential trend (*P* = 0.085).

Table 1. Socio-demographic Characteristics of Suspected AWD Cases Reported at Cumilla General Hospital.

**Table 1:** Socio-demographic characteristics of suspected acute watery diarrhea cases reported at Cumilla General Hospital.

Variable	Category	N (%)	RDT-negative (N = 928)	RDT-positive (N = 49)	*P*-value
Age group	<5 years	548 (56.09%)	526 (56.68)	22 (44.90)	0.404
	5-17 years	62 (6.35%)	59 (6.36)	3 (6.12)	
	18-45 years	270 (27.64%)	251 (27.05)	19 (38.78)	
	46-60 years	63 (6.45%)	59 (6.36)	4 (8.16)	
	≥61 years	34 (3.48%)	33 (3.56)	1 (2.04)	
Gender	Male	514 (52.61%)	487 (52.48)	27 (55.10)	0.72
	Female	463 (47.39%)	441 (47.52)	22 (44.90)	
Occupation^a^	Service holder	34 (9.26%)	30 (8.75)	4 (16.67)	0.589
	Housewife	217 (59.13%)	203 (59.18)	14 (58.33)	
	Unemployed	29 (7.90%)	28 (8.16)	1 (4.17)	
	Laborer	75 (20.44%)	70 (20.41)	5 (20.83)	
	Students	12 (3.27%)	12 (3.50)	0 (0.00)	
Educational status^a^	No formal education	137 (37.33%)	133 (38.78)	4 (16.67)	0.085
	Below secondary	116 (31.61%)	105 (30.61)	11 (45.83)	
	Secondary and above	114 (31.06%)	105 (30.61)	9 (37.50)	

a Occupation and education variables are reported for adult participants only (≥18 years; N = 367). All other variables are calculated using the full sample (N = 977). Percentages are based on the respective denominators.

None of the socio-demographic characteristics, including age, gender, occupation, or education, showed no clear evidence of association based on the estimated effects (*P*-values > 0.05). However, observed trends such as a higher proportion of positive cases among adults aged 18-45 years and service holders may indicate contextual or behavioral risk patterns that merit further exploration in future studies

Table 2. Association between environmental, behavioral, and household risk factors and RDT positivity among suspected Acute Watery Diarrhoea (AWD)

**Table 2:** Association between environmental, behavioral, and household risk factors and rapid diagnostic test positivity among suspected acute watery diarrhea.

Variable	Category	N (%)	RDT-negative (N = 928)	RDT-positive (N = 49)	*P*-value
Water source	Tap water (Yes)	897 (91.81%)	852 (91.81%)	45 (91.84%)	0.995
	Tube well (Yes)	132 (13.51%)	125 (13.47%)	7 (14.295)	0.871
	Bottled water (Yes)	1 (0.1%)	1 (0.11%)	0 (0)	0.818^a^
Intake water form	Untreated (Yes)	976 (99.9%)	927 (99.89%)	49 (100%)	0.818
	Boiled (Yes)	29 (2.97%)	27 (2.91%)	2 (4.08%)	0.638^a^
	Filtered (Yes)	7 (0.72%)	7 (0.75%)	0 (0)	0.542^a^
Take food from roadside vendors (Yes)		659 (67.45%)	626 (67.46%)	33 (67.35%)	0.987
Take food at any large gatherings (Yes)		2 (0.2%)	1 (.11%)	1 (2.04%)	0.004^a^
Household members suffer from the same disease (Yes)		9 (0.92%)	6 (0.65%)	3 (6.12%)	0.000^a^
Neighbors have the same disease (Yes)		16 (1.64%)	14 (1.51%)	2 (4.08%)	0.167
Washing habit after defecation	Yes	976 (99.9%)	927 (99.89%)	49 (100%)	0.818
	No	977 (100%)	1 (.11%)	0 (0)	
Take antibiotics for current illness	Yes	881 (90.17%)	838 (90.30%)	43 (87.76%)	0.559
	No	96 (9.83%)	90 (9.7%)	6 (12.24%)	

Abbreviations: AWD, acute watery diarrhea; RDT, rapid diagnostic test.

a Occupation and education variables are reported for adult participants only (≥18 years; N = 367). All other variables are calculated using the full sample (N = 977). Percentages are based on the respective denominators.

Among 977 suspected AWD cases, no clear associations were observed based on the estimated effects between RDT outcomes and most environmental, behavioral, or hygiene-related factors such as drinking tap water (≈91.8%), consuming untreated water (≈99.9%), eating roadside food (≈67%), or sanitation practices (≈100%). However, consumption of food at large gatherings (although rare, 0.2%; *P* = 0.004) and household clustering of illness (6.1% vs 0.65%; *P* < 0.001) showed higher occurrence among RDT-positive cases. Two culture-positive cases were RDT-negative.

The environmental and behavioral variables, including untreated water consumption and handwashing practices, were highly homogeneous across the study population. Because of the absence of a comparison group (e.g. individuals practicing water treatment or consistent soap use), these variables could not be meaningfully analyzed as independent risk factors. Instead, they reflect prevailing baseline vulnerabilities that may facilitate the transmission of AWD in the study setting.

### Clinical characteristics of RDT-positive AWD cases

Among the 49 RDT-positive suspected AWD cases, 95.92% presented with loose watery stools, and 4.08% with rice-water stools. Regarding dehydration, 95.92% had some dehydration and 4.08% had severe dehydration, with no cases of patients without dehydration. Abdominal cramping and vomiting were present in all RDT-positive cases (100%), while 97.96% experienced fever. These findings highlight the consistent clinical profile of AWD among RDT-positive cases and underscore the importance of rapid clinical recognition and timely management.

### Trends of AWD cases based on months and years

The analysis of suspected and RDT-positive cases from June 2022 to September 2024 reveals distinct trends over the months and years. Initially, suspected cases fluctuated, with a notable peak in December 2022 at 42 cases, followed by a gradual decline in early 2023. A resurgence was observed in late 2023, particularly in November, which recorded the highest number of suspected cases at 53. This upward trend continued into early 2024, with January 2024 also showing a significant count of 56 suspected cases. However, a decline was noted in the subsequent months, with zero suspected cases reported in May and June 2024 in the facility records.

**Figure 1:**
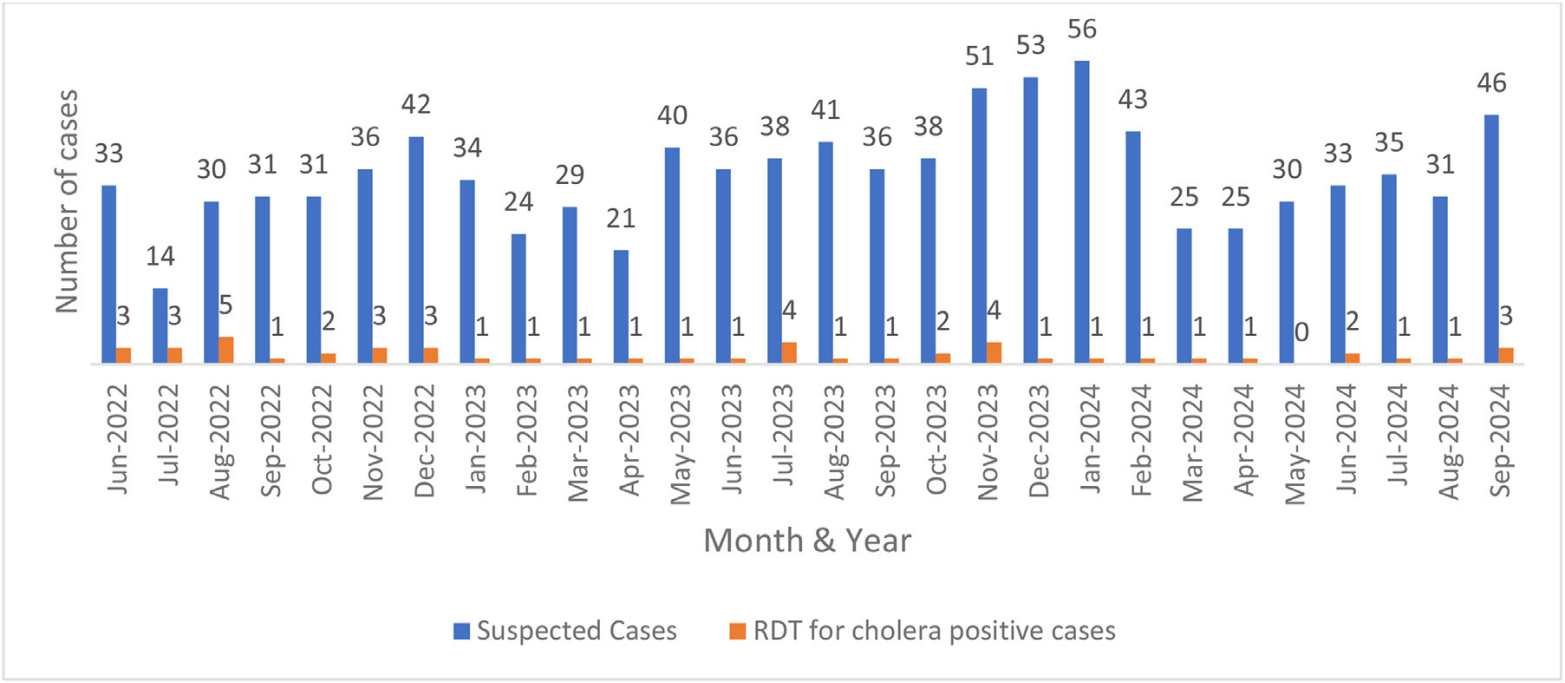
Monthly trends of suspected acute watery diarrhea cases and rapid diagnostic test-positive cholera cases at Cumilla General Hospital, June 2022-September 2024.

Abbreviations: AWD, acute watery diarrhea; RDT, rapid diagnostic test.

RDT-positive cases remained consistently low throughout the period, with a maximum of five cases in August 2022 and occasional increases. The suspected cases exhibited more variability, RDT-positive cases did not show a similar pattern of increase. In summary, this indicates a general increase in suspected cases over the observed period, particularly in late 2023 and early 2024, while RDT-positive cases remained relatively stable and low (Figure 1).

### Seasonal trend of suspected AWD cases

The line diagram shows the following seasonality pattern (month-wise) of suspected AWD cases: Pre-monsoon months (July-August) showed low suspected cases, with a slight increase in 2023-24. Monsoon months (September-November) experienced significant peaks, particularly in November, with suspected cases reaching 53 in 2023-2024. Post-monsoon (December-February) saw peaks in suspected cases, notably 56 in January 2023-2024 (Figure 3).

### Seasonal trend of RDT for cholera-positive AWD cases

**Figure 2:**
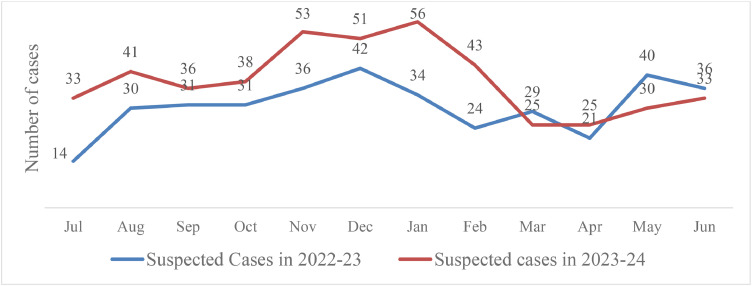
Seasonal distribution of suspected acute watery diarrhea cases by month across 2022-2024.

Abbreviation: AWD, acute watery diarrhea.

**Figure 3:**
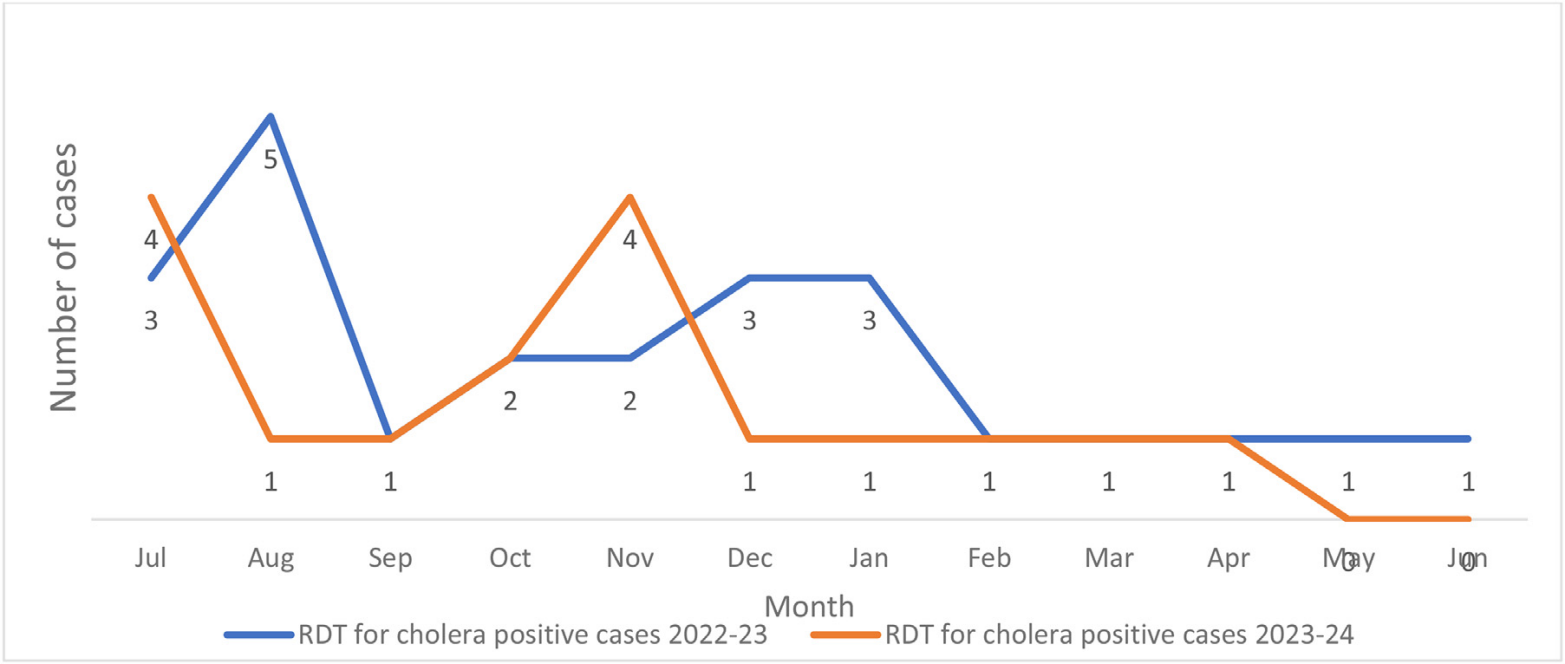
Seasonal distribution of rapid diagnostic test-positive cholera cases by month across 2022-2024.

Abbreviations: AWD, acute watery diarrhea; RDT, rapid diagnostic test.

The line diagram shows the following seasonality pattern (month-wise) of RDT-positive cases: RDT-positive cases exhibit a slight increase in July followed by a decline in August (Figure 2). RDT-positive cases demonstrate a more stable low incidence from September to March. The trend shows a decline of RDT-positive cases to zero by May and June of 2023-24. RDT-positive cases remained low but followed a similar trend, peaking at four in November (Figure 3).

Given the reliance on RDT without routine laboratory confirmation through culture/PCR, these findings should be interpreted as indicative of suspected cholera trends rather than definitive incidence.

## Discussion

This study provides a detailed description of AWD surveillance at Cumilla General Hospital, Bangladesh, with emphasis on epidemiological profile and demographics of cholera. A recent diarrhea outbreak in Chittagong overwhelmed local hospitals, highlighting the strain of acute diarrheal diseases on health systems in Bangladesh [16]. The seasonality and geographic clustering of AWD cases are evident from the surveillance data, consistent with our understanding of the epidemiology of cholera in Bangladesh. The peaks observed in suspected cases during the monsoon and post-monsoon seasons. The pattern receives its seasonality owing to environmental factors, such as upsurge of water contamination during heavy rainfall. The concentrated cases in Cumilla Adarsha Sadar indicate the necessity for time-bound, targeted public health measures in known hot spots or high-risk areas. These findings are consistent with previous research highlighting the influence of increased urbanization, inadequate sanitation, and low water quality on cholera transmission [10], [17].

Strengthening diagnostic confirmation at sentinel sites, particularly through routine use of culture, is essential. This aligns with the findings of national sentinel surveillance conducted between 2014 and 2021, which showed that culture-positive cholera cases accounted for only 5.2% of all suspected cases, underscoring the pressing need for stronger diagnostic coverage and consistency across sentinel sites [18]. The results also reaffirm the significant burden of rotavirus among Bangladeshi children aged <5 years, particularly during the post-monsoon period [19]. The most suspected cases were in children aged <5 years with a high level of vulnerability. Drinking from contaminated sources showed higher odds of contracting cholera. Poor handwashing, even with access to shared latrines, remains a major gap. Some RDT-positive cases had rice-water stool and severe dehydration, underscoring the need for early recognition and treatment. Detected *Vibrio cholerae* risk factors align with recent genomic studies, and the high pre-hospital antibiotic use (90%) raises concern about misuse and rising antimicrobial resistance. Similar patterns have been observed in other regions, where extreme weather events, such as cyclones have triggered large-scale cholera outbreaks, further exposing weaknesses in WASH infrastructure and emergency preparedness systems [20].

Integration of AMR surveillance into AWD surveillance could allow for early warning of resistance trends. They are difficult to treat strains and emphasize the necessity of incorporating molecular diagnostics into surveillance practices [21]. In addition, the contribution of biofilm-bound V. cholerae populations in estuaries and their interaction with lytic phages will need to be investigated for developing more accurate control models [22]. The strengths and limitations of the surveillance system include timeliness and representativeness of the sentinel AWD surveillance system. Providing demographic and clinical variables increases the scale of insights, facilitating tailored response. The incorporation of the system into national public health structure facilitates efficient communication with stakeholders, thereby supporting prompt decision-making. Given the seasonal spikes and the observed surge following flood events, integrating AWD surveillance and early warning systems into broader climate adaptation and disaster risk management frameworks is essential for anticipatory public health responses [23]. Lessons from other infectious disease outbreaks emphasize the importance of early surveillance, coordinated response mechanisms, and strong public health preparedness systems to effectively manage emerging threats [24]. The use of RDT as the primary diagnostic tool may have introduced misclassification bias because of its variable sensitivity and specificity. While RDT is useful for rapid field-level screening, it cannot replace culture or PCR for definitive confirmation.

### Limitations of the study

This study has several limitations. The broad case definition may allow other pathogens and co-infections with *Vibrio cholerae*, leading to possible misclassification. The use of passive surveillance may underestimate AWD cases, especially among those with mild or asymptomatic illnesses. Diagnostic sensitivity issues and data quality variability may impact findings. The absence of culture confirmation in 84% of RDT-positive cases reflects limited diagnostic capacity and may affect case classification. A key limitation of this study is the reliance on RDT without routine microbiological confirmation. This may lead to both false positive and false negative classifications, affecting the accuracy of cholera burden estimation. As a result, the study findings should be interpreted as indicative of AWD trends with limited inference on cholera incidence. Several environmental and behavioral variables showed minimal variation across participants, limiting their utility for inferential analysis. The absence of a comparison group restricted the ability to identify clear associations, and these variables were therefore interpreted as baseline vulnerabilities rather than causal risk factors. Additionally, not all cases of AWD are attributable to cholera, as other pathogens such as rotavirus and enterotoxigenic Escherichia coli may contribute to the observed burden, which may lead to potential overestimation of cholera-specific interpretations.

## Conclusion

This study demonstrates a substantial burden of AWD in a hospital-based surveillance setting, while only a small proportion of cases (≈5%) tested positive for cholera by RDT. Strengthening early warning systems, surveillance integration, and coordinated response frameworks will be critical for improving outbreak preparedness and resilience in Bangladesh [6], [25]. These findings suggest that cholera represents a limited share of AWD cases in this dataset and should be interpreted cautiously given diagnostic constraints and the absence of routine laboratory confirmation.

## Declaration of competing interest

The authors declare that there are no conflicts of interest related to this study.

## References

[1] Ali M., Nelson A.R., Lopez A.L., Sack DA. (2015). Updated global burden of cholera in endemic countries. PLoS Negl Trop Dis.

[2] Kosek M., Bern C., Guerrant RL. (2003). The global burden of diarrhoeal disease, as estimated from studies published between 1992 and 2000. Bull World Health Organ.

[3] Chowdhury F., Khan I.A., Patel S., Siddiq A.U., Saha N.C., Khan A.I. (2015). Diarrheal illness and healthcare seeking behavior among a population at high risk for diarrhea in Dhaka, Bangladesh. PLoS One.

[4] Liu L., Johnson H.L., Cousens S., Perin J., Scott S., Lawn J.E. (2012). Global, regional, and national causes of child mortality: an updated systematic analysis for 2010 with time trends since 2000. Lancet.

[5] United Nations Children’s Fund (2021).

[6] World Health Organization. Diarrhoeal disease: key facts. https://www.who.int/news-room/fact-sheets/detail/diarrhoeal-disease; 2023. [accessed 12 June 2026]

[7] Islam M.T., Khan A.I., Khan Z.H., Tanvir N.A., Ahmmed F., Afrad M.M.H. (2021). Acute watery diarrhea surveillance during the Rohingya crisis 2017-2019 in Cox’s Bazar, Bangladesh. J Infect Dis.

[8] International Centre for Diarrhoeal Disease Research, Bangladesh. Acute Watery Diarrhoea Surveillance Reports. https://www.icddrb.org; 2021. [accessed 12 June 2026]

[9] Das S.K., Begum D., Ahmed S., Ferdous F., Farzana F.D., Chisti M.J. (2014). Geographical diversity in seasonality of major diarrhoeal pathogens in Bangladesh observed between 2010 and 2012. Epidemiol Infect.

[10] Chowdhury F., Rahman M.A., Begum Y.A., Khan A.I., Faruque A.S.G., Saha N.C. (2011). Impact of rapid urbanization on the rates of infection by Vibrio cholerae O1 and enterotoxigenic Escherichia coli in Dhaka, Bangladesh. PLoS Negl Trop Dis.

[11] Khan A.I., Islam M.T., Amin M.A., Khan Z.H., Qadri F. (2023). Outbreak of diarrheal diseases causes mortality in different geographical locations of Bangladesh during the 2021 COVID-19 era. Front Public Health.

[12] Tauheed I., Ahmed T., Akter A., Firoj M.G., Ahmmed F., Rahman S.I.A. (2023). A snap-shot of a diarrheal epidemic in Dhaka due to enterotoxigenic Escherichia coli and Vibrio cholerae O1 in 2022. Front Public Health.

[13] Uwishema O., Masunga D.S., Naisikye K.M., Bhanji F.G., Rapheal A.J., Mbwana R. (2023). Impacts of environmental and climatic changes on future infectious diseases. Int J Surg.

[14] World Health Organization (2017). Ending cholera: a global roadmap to 2030. https://www.gtfcc.org/wp-content/uploads/2020/09/ending-cholera-a-global-roadmap-to-2030.pdf.

[15] Arnaout A.Y., Nerabani Y., Sawas M.N., Alhejazi T.J., Farho M.A., Arnaout K. (2024). Acute watery diarrhoea cases during cholera outbreak in Syria: a cohort study. BMJ Open.

[16] Dhaka Tribune. Chittagong hospitals overwhelmed by diarrhoea outbreak.https://www.dhakatribune.com/bangladesh/310403/chittagong-hospitals-overwhelmed-by-diarrhoea; 2023. [accessed 12 June 2026]

[17] Emch M., Yunus M., Escamilla V., Feldacker C., Ali M. (2010). Local population and regional environmental drivers of cholera in Bangladesh. Environ Health.

[18] Islam M.T., Hegde S.T., Khan A.I., Bhuiyan M.T.R., Khan Z.H., Ahmmed F. (2023). National hospital-based sentinel surveillance for cholera in Bangladesh: epidemiological results from 2014 to 2021. Am J Trop Med Hyg.

[19] Ngo T.H., Tran H.T., Vu H.D., Phan T.N., Tran Q.T., Doan Y.H. (2020). Rotavirus infection and genotype distribution among children with acute diarrhea in Vietnam, 2012-2015. J Infect Public Health.

[20] Uwishema O., Elebesunu E.E., Bouaddi O., Kapoor A., Akhtar S., Effiong F.B. (2022). Poliomyelitis amidst the COVID-19 pandemic in Africa: efforts, challenges and recommendations. Clin Epidemiol Glob Health.

[21] Monir M.M., Islam M.T., Mazumder R., Mondal D., Nahar K.S., Sultana M. (2023). Genomic attributes of Vibrio cholerae responsible for a massive cholera outbreak in Bangladesh. Nat Commun.

[22] Sultana M., Nusrin S., Hasan N.A., Sadique A., Ahmed K.U., Islam A. (2018). Biofilms comprise a component of the annual cycle of Vibrio cholerae in the Bay of Bengal estuary. mBio.

[23] United Nations Children’s Fund. Climate crisis is a child rights crisis: introducing the Children’s Climate Risk Index. https://www.unicef.org/reports/climate-crisis-child-rights-crisis; 2022. [accessed 12 June 2026]

[24] Focus Adriano L., Nazir A., Uwishema O. (2023). The devastating effect of cyclone Freddy amidst the deadliest cholera outbreak in Malawi: a double burden for an already weak healthcare system - short communication. Ann Med Surg.

[25] Uwishema O., Nnagha E.M., Chalhoub E., Nchasi G., Mwazighe R.M., Akin B.T. (2021). Dengue fever outbreak in Cook Island: A rising concern, efforts, challenges, and future recommendations. J Med Virol.

